# Cyr61/CCN1 signaling is critical for epithelial-mesenchymal transition and stemness and promotes pancreatic carcinogenesis

**DOI:** 10.1186/1476-4598-10-8

**Published:** 2011-01-13

**Authors:** Inamul Haque, Smita Mehta, Monami Majumder, Kakali Dhar, Archana De, Douglas McGregor, Peter J Van Veldhuizen, Sushanta K Banerjee, Snigdha Banerjee

**Affiliations:** 1 Cancer Research Unit, Veterans Affairs Medical Center, Kansas City, MO, and Division of Hematology and Oncology, Department of Medicine, USA; 2 Department of Pathology, University of Kansas Medical Center, Kansas City, Kansas, USA; 3 Department of Anatomy and Cell Biology, University of Kansas Medical Center, Kansas City, Kansas, USA

## Abstract

**Background:**

Despite recent advances in outlining the mechanisms involved in pancreatic carcinogenesis, precise molecular pathways and cellular lineage specification remains incompletely understood.

**Results:**

We show here that Cyr61/CCN1 play a critical role in pancreatic carcinogenesis through the induction of EMT and stemness. Cyr61 mRNA and protein were detected in the early precursor lesions and their expression intensified with disease progression. Cyr61/CCN1 expression was also detected in different pancreatic cancer cell lines. The aggressive cell lines, in which the expressions of mesenchymal/stem cell molecular markers are predominant; exhibit more Cyr61/CCN1 expression. Cyr61 expression is exorbitantly higher in cancer stem/tumor initiating Panc-1-side-population (SP) cells. Upon Cyr61/CCN1 silencing, the aggressive behaviors are reduced by obliterating interlinking pathobiological events such as reversing the EMT, blocking the expression of stem-cell-like traits and inhibiting migration. In contrast, addition of Cyr61 protein in culture medium augments EMT and stemness features in relatively less aggressive BxPC3 pancreatic cancer cells. Using a xenograft model we demonstrated that cyr61/CCN1 silencing in Panc-1-SP cells reverses the stemness features and tumor initiating potency of these cells. Moreover, our results imply a miRNA-based mechanism for the regulation of aggressive behaviors of pancreatic cancer cells by Cyr61/CCN1.

**Conclusions:**

In conclusion, the discovery of the involvement of Cyr61/CCN1 in pancreatic carcinogenesis may represent an important marker for PDAC and suggests Cyr61/CCN1 can be a potential cancer therapeutic target.

## Background

Pancreatic ductal adenocarcinoma (PDAC) is the tenth most common cancer diagnosed in the United States and fourth most common cause of cancer death in the United States. The five year survival rate for patients with pancreatic adenocarcinoma is approximately 5% [1] with a median survival rate of 6 months or less [2]. Although improvement is being made through the development of targeted therapies [3], the prognosis and treatment of PDAC is still unsatisfactory. This is due both to the late presentation and the lack of an effective treatment strategy [2]. Therefore, there is a growing need to understand of the mechanism(s) in the progression of pancreatic adenocarcinoma which will ultimately lead to an improvement of treatment strategies for this devastating disease.

Cyr61 (cysteine-rich 61) is a member of the CCN family of growth factors that includes CTGF, NOV, WISP-1, WISP-2 and WISP-3 [4]. It is a 42 kDa secreted, growth factor-inducible immediate-early response gene [5]. Like other members of CCN-family, Cyr61 contains four different conserved molecular domains. These include insulin-like growth factor-binding protein (IGFBP), the von Willebrand factor type C repeat, the thrombospondin type 1 repeat (TSP-1) and Carboxyl termini of several extracellular proteins (CT) [4]. Cyr61 is known to link cell surface and extracellular matrix and plays important roles on cell adhesion, proliferation, migration, differentiation and angiogenesis during normal developmental and pathophysiological processes [4]. Except for lung cancers [6], endometrial cancers [7] and leiomyomas [8], the level of cyr61 expression has been found to be increased in various human cancers including breast, rhabdomyosarcomas, melanomas, gliomas, gastric, colon, bladder papillomas and prostate cancers[9-13]. Over production of Cyr61 may play a critical role in the development and progression of these cancers; possibly through integrin-linked kinase signal-networking [13-15]. In addition, Cyr61 has been shown to promote invasion and metastasis of tumors growing in preirradiated stroma [16]. Although its role in PDAC still remains poorly understood, recent evidence showed that Cyr61 expression was increased in metastatic lesions in a clinically relevant model of pancreatic adenocarcinoma and suggested that the interaction between Cyr61 and αvβ3 may promote the formation of peritoneal metastases [17].

To establish whether Cyr61 is indeed a critical signaling factor in PDAC, we have studied the expression profile of Cyr61 in human pancreatic adenocarcinoma samples and different cell lines at protein and mRNA levels; and determined its functional role in the development and progression of pancreatic adenocarcinoma by silencing Cyr61 retrovirally or exposing cells to recombinant Cyr61 protein. The studies clearly implicate Cyr61 as an important factor in determining PDAC aggressiveness as it promotes epithelial to mesenchymal transition (EMT), tumor stemness, *in vitro *migration and tumorigenicity in xenograft model, possibly through the regulation of multiple miRNAs that are known to link with the progression of cancers and survival and the maintenance of cancer stem cells [18,19]. Cyr61 could therefore represent an ideal target in PDAC therapy.

## Results

### Cyr61/CCN1 is differentially expressed in pancreatic tissue samples

**Figure 1:**
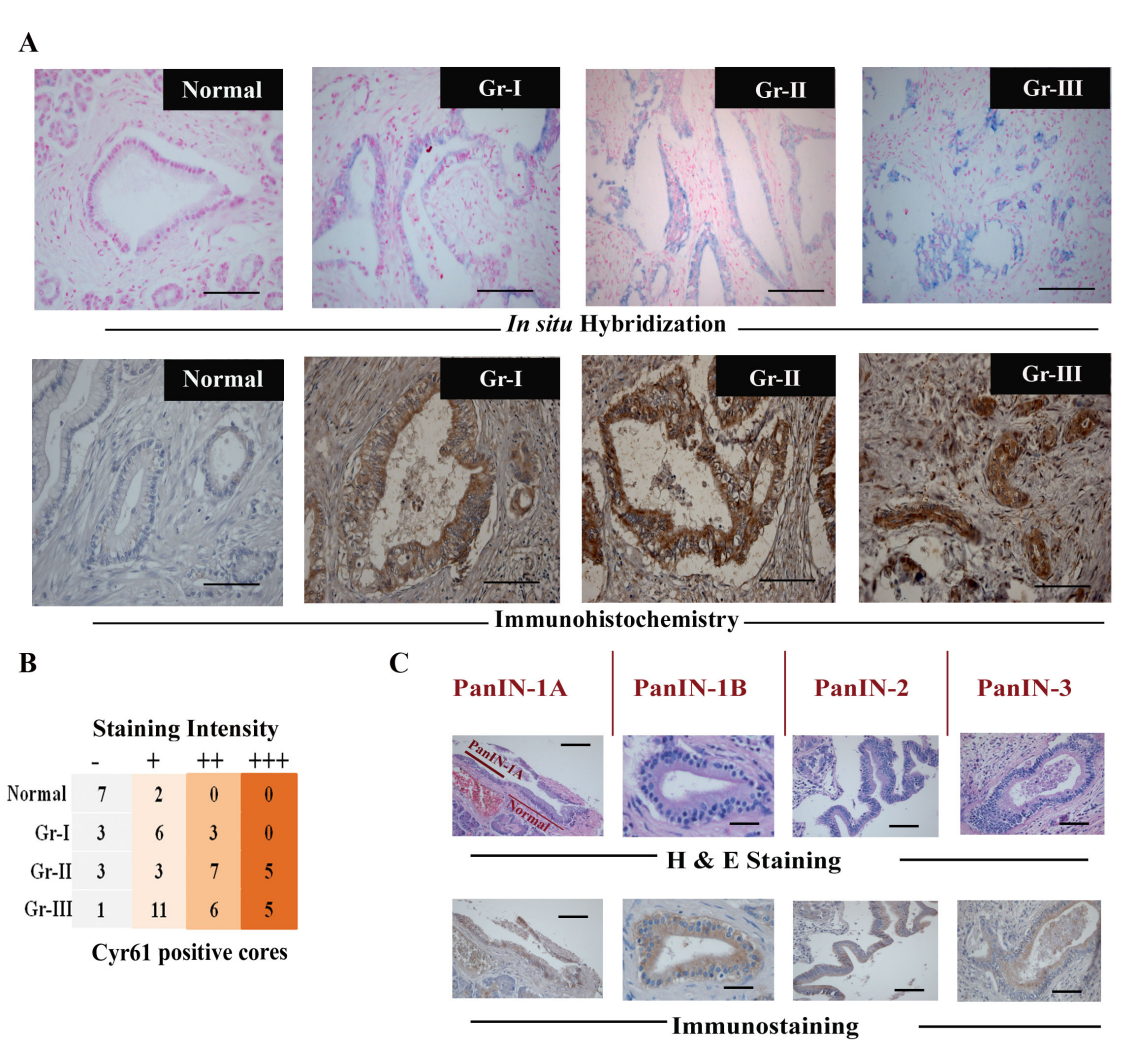
**Differential expression of Cyr61 mRNA and protein in Normal pancreas and pancreatic ductal adenocarcinoma tissue samples**. **A**. The photomicrographs (upper panel and lower panel) represents the distribution pattern of Cyr61 mRNA (blue color) by *in situ hybridization *using digoxigenin (DIG)-labeled PCR generated Cyr61 specific non-radioactive probe and Cyr61 protein (brown color) by immunohistochemistry in a tissue array containing adjacent normal pancreas, and different grades of ductal adenocarcinoma (Gr-I, Gr-II and Gr-III) respectively. Scale bars: 100μm. **B**. Distribution profile and labeling intensity of Cyr61 protein in PDAC tissue samples. Gr-I, Grade 1, Gr-II, Grade II and Gr-III, Grade III. (-), No stain, (+), light brown, (++), moderately dark brown, and (+++), dark brown. **C**. H & E staining and immunohistochemical identification of Cyr61 in precursor lesions of pancreatic cancers. Scale bars: 50μm.

To determine the status of Cyr61 mRNA in PDAC, we evaluated high grade primary pancreatic adenocarcinoma tissue samples (N = 16) along with adjacent normal pancreas. We found ~81% (13 out of 16) pancreatic cancer specimens exhibited over-expression of Cyr61 mRNA as compared to adjacent normal samples where expression was either undetected or minimal. The distribution of Cyr61 mRNA was mainly in the infiltrating ducts and acini of the tumor area **(Figure **1A, **upper panel)**. To validate the results of *in situ *hybridization, we then determined Cyr61 protein status in PDAC by immunohistochemistry using a tissue array slide and a Cyr61 specific antibody. Each slide contained 63 specimens and these included: ductal adenocarcinoma Grade I (N = 10), Grade II (N = 15) and Grade III (N = 23) in addition to normal adjacent pancreas (N = 3), chronic pancreatitis *(*N = 3), mucus and digestive tumor cells (N = 2), islet cell carcinoma *(*N = 6), fibrous tissue and fatty tissue (N = 1). Data on chronic pancreatitis, mucinous and islet cell carcinoma were excluded from this study. We found ~85% (53 out of 63) PDAC samples were Cyr61 positive and the level of Cyr61 protein was markedly higher in PDAC specimens as compared to adjacent normal tissues where its expression was minimal **(Figures **1A**lower panel and 1B)**. Cyr61 is distributed in the cytoplasm of tumor cells of the infiltrating pancreatic ducts and acinar cells. The intensity of the staining increased markedly as the disease progressed from Grade-I to Grade III. However, the expression profile was not grade-dependent **(Figures **1A**and **1B**)**. Moreover, increased level of Cyr61 protein was also detected in histologically defined precursor lesions (PanINs; PanIN-1A-PanIN-3) **(Figure **1C**)**.

### Cyr61/CCN1 expression in pancreatic adenocarcinoma cell lines at mRNA and protein level

**Figure 2:**
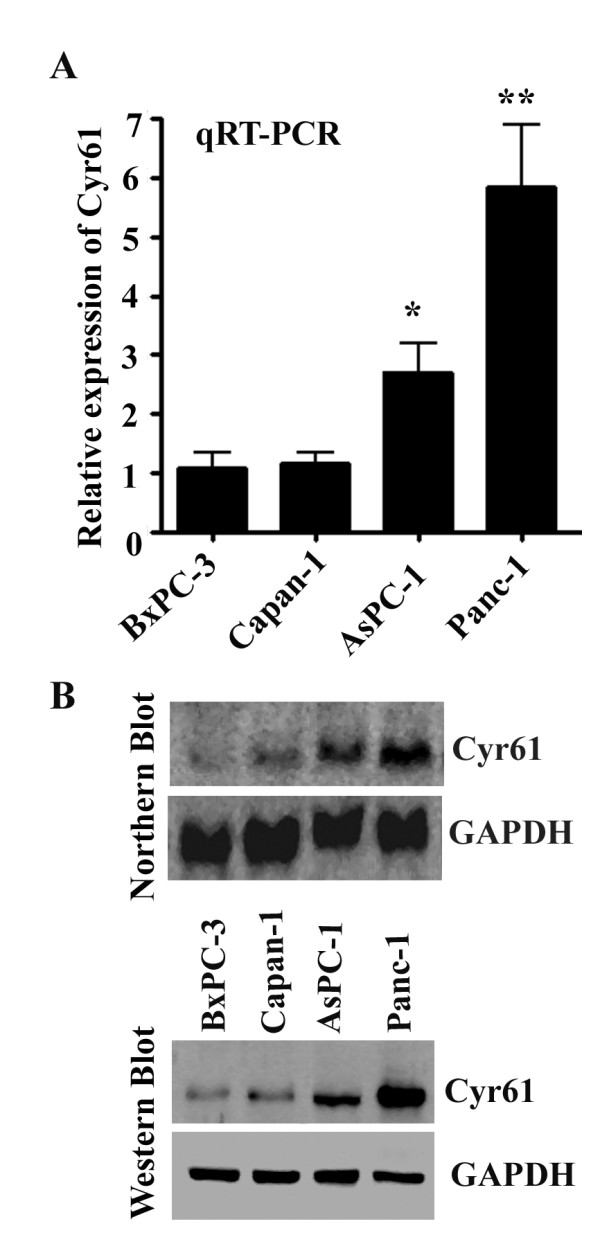
**Expression of Cyr61 in different pancreatic cancer cell lines**. **A**. The bar graph indicates the quantitative relative expression of Cyr61 in different pancreatic cancer cell lines by real time PCR. **B**. Upper panel: Northern blot analysis of Cyr61 in different pancreatic cancer cell lines using a nonradioactive digoxigenin-labeled, PCR-generated Cyr61 specific probe. GAPDH was used as a control probe to eliminate the loading differences. Lower panel: Immuno-western blotting of Cyr61 in different pancreatic cancer cell lines.

Our next goal was to determine the status of Cyr61 mRNA and protein in different pancreatic cancer cell lines. These included BxPC-3, Capan-1, Aspc-1, and Panc-1. These cells were well-characterized from less aggressive (i.e. BXPC-3 and Capan-1) to highly aggressive cell lines (i.e. Aspc-1, and Panc-1) with varied degrees of EMT markers [20]. Quantitative real-time PCR, Northern blotting and Western blotting analysis revealed that Cyr61 mRNA and protein were detected in BxPC-1, Capan-1, AsPC-1 and Panc-1 with varying degrees of expression **(Figures **2A**and **2B**)**. The highest expression of RNA and protein was detected in Panc-1 cells followed by AsPC-1, Capan-1 and BxPC-3 **(Figure **2**)**.

### Suppression of Cyr61/CCN1 inhibits in vitro migration of pancreatic cancer cells

**Figure 3:**
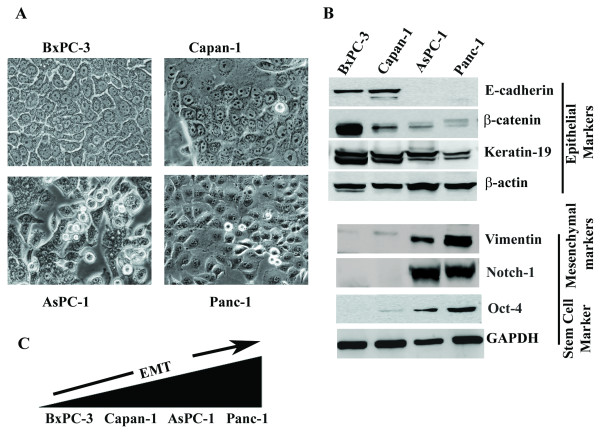
**Comparison of morphologies and expressions of EMT and stem cell markers in different pancreatic cancer cells**. **A**. Photo-micrographs of the morphology of different pancreatic cancer cells growing at low density on tissue culture plastic. **B**. Western blots analysis of various epithelial, mesenchymal and stem cell markers. **C**. Diagrammatic illustration of aggressive/metastatic phenotypes of different pancreatic cancer cells.

**Figure 4:**
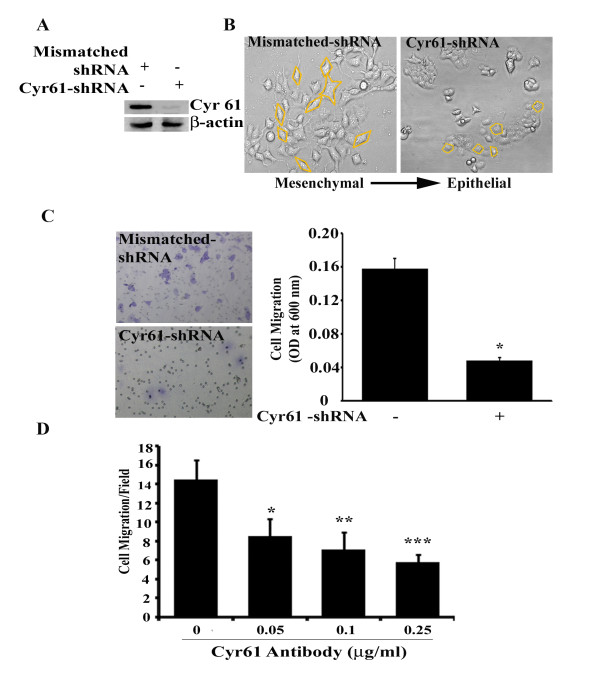
**Silencing of Cyr61 promotes mesenchymal to epithelial transition (MET) and blocks *in vitro *migration of Panc-1 cells**. **A**. Western blot illustrating shRNA mediated inhibition of Cyr61 expression in Panc-1 cells. **B**. The photomicrographs represent the morphological changes (from mesenchymal to epithelial transition, MET) in mismatched and Cyr61 silenced Panc-1 cells. The morphology of each cells are marked with yellow lines.**C**. The photomicrographs represent the difference in the *in vitro *migration of mismatched and Cyr61 silenced Panc-1 cells toward the serum for 24 hours. Results are averages ± SEM (n = 6). *p < 0.001 *vs *mismatched. **D**. Demonstration of the inhibition of paracrine action of Cyr61 on migration toward the serum for 48 h by different doses of Cyr61 neutralizing antibody. Results are averages ± SEM (n = 6). *p < 0.05 *vs *vehicle treated control; **p < 0.01 *vs *vehicle treated control and ***p < 0.001 *vs *vehicle treated control.

To investigate the pathobiological role of Cyr61 in pancreatic cancer, first, we determined the morphology as well as the status of epithelial and mesenchymal/stem cell markers in BxPC-3, Capan-1, AsPC-1 and Panc-1. Consistent with previous work [20], we found that BxPC-3 and Capan-1 cells are morphologically epithelial in nature (**Figure **3A**)**, but these cells differentially express both epithelial (i.e., Keratin-19, E-cadherin and β-catenin) and mesenchymal (i.e., Vimentin) markers (**Figure **3B**)**. In contrast, AsPC-1 and Panc-1 cells are mixed populations of epithelial and spindle-shaped mesenchymal type cells along with stemness and express epithelial, mesenchymal and stem cell markers with some exclusion in Panc-1 cells. These cells express only Keratin-19 and high levels of Vimentin, Notch-1 and Oct-4 (**Figure **3B**)**. E-cadherin and β-catenin expression was undetected or minimally detected in Panc-1 cells and AsPC-1 cells. Taken together, these and previous studies suggest that these pancreatic cancer cell lines are a phenotypically mixed population but not identical because they express epithelial and mesenchymal markers with varying degrees and their aggressive/metastatic behaviors are different. Therefore, these cells can be categorized from less aggressive (i.e., BxPC-3 and Capan-1 cell lines) to highly aggressive (i.e., AsPC-1 and Panc-1) with varied degrees of epithelial-mesenchymal transition (EMT) and stemness (**Figure **3C**)**. Next, we attempted to determine the role of Cyr61 in the morphological and behavioral alterations of pancreatic cancer cells by focusing on *in vitro *migration. To do so, we blocked the Cyr61 expression in Panc-1 cells by stable transfection of a pSilencer 5.1-U6-retroviral vector-containing Cyr61-specific shRNA, and we evaluated the expression of Cyr61 in these cells. We found that more than 95% of Cyr61's expression was suppressed by stable transfection of Cyr61-shRNA while this effect was undetected in mismatched-shRNA transfected cells **(Figure **4A**)**. After confirmation, the morphology of Cyr61 positive **(Panc-1**^**Cyr61+**^**) **and Cyr61 knockout **(Panc-1**^**KOCyr61**^**) **cells was evaluated. We found that the morphology of the Panc-1 cells was markedly altered with a transition from the mesenchymal/fibroblast-type to the epithelial-type **(Figure **4B**)**. Finally, we determined whether Cyr61 has any role in *in vitro *migration of these cells. To do so, we seeded Panc-1^Cyr61+ ^and Panc-1^KOCyr61 ^cells in the upper chamber of the Boyden chamber to test *in vitro *migration toward the serum for 24 h. We stained the migrated cells with Crystal violet, and then performed a colorimetric (quantitative) analysis using an ELISA plate reader. This study reveals that the migration of Panc-1^KOCyr61 ^cells toward the serum was significantly less as compared to Panc-1^Cyr61+ ^cells **(Figure **4C**)**. The results were consistent when the paracrine action of Cyr61 was blocked by adding a human polyclonal anti-rabbit Cyr61 blocking antibody in the media **(Figure **4D**)**. We repeated the experiments in AsPC-1 cells and also found that the silencing of Cyr61 alters the morphology of AsPC-1 cells as well as significantly blocks the *in vitro *migration of these cells (data not shown). Finally, we evaluated the impact of Cyr61 on pancreatic cancer cell proliferation. We found that blocking Cyr61 in Panc-1 cells by shRNA or a Cyr61-specific antibody has no effect on the proliferation of Panc-1 cells (data not shown).

### Reverse EMT (epithelial-mesenchymal transition) by blocking Cyr61/CCN1 expression

**Figure 5:**
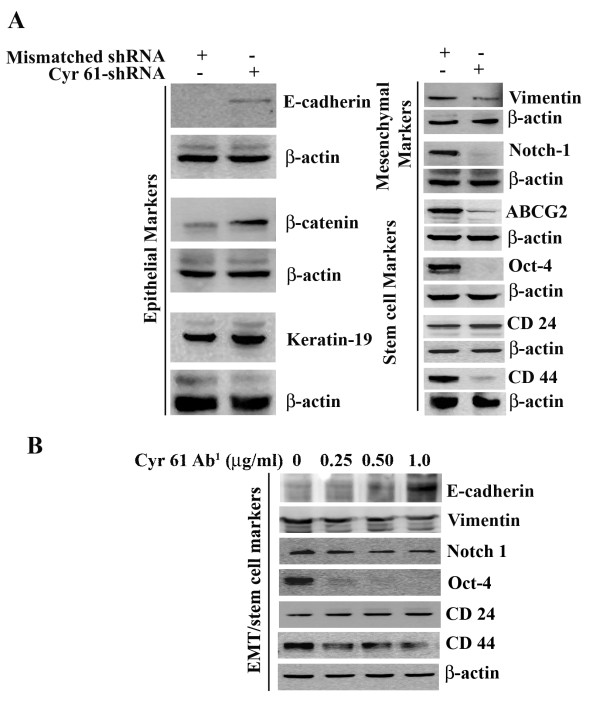
**Cyr61 silencing by RNA*i *(shRNA) (A) or blocking its paracrine activities by a Cyr61 neutralizing antibody (B) modulates the expressions of different epithelial and mesenchymal/stemness markers in Panc-1 cells**. Equal amount of cell lysates were analyzed by Western blotting using specific antibodies. β-actin antibody was taken as a loading control.

**Figure 6:**
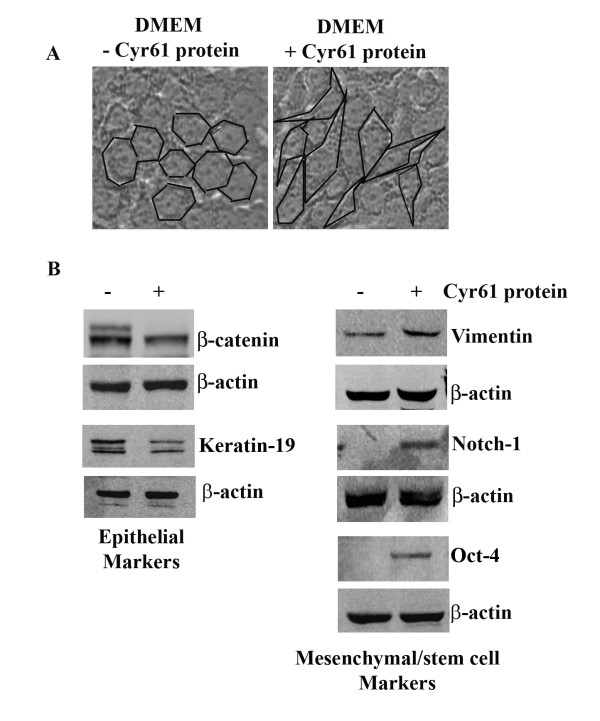
**Induction of EMT and stemness by Cyr61 recombinant protein in Cyr61 negative BxPC-3 cells**. **A**. The photomicrographs represent the morphological changes in BxPC-3 cells growing in the presence and absence of recombinant Cyr61 protein (100 ng/ml) for 48 h. The morphology of each cell is marked with black lines. **B**. Western blot analysis of various epithelial, mesenchymal and stem cell markers.

An EMT event is involved in the formation of motile cells from parent epithelial cells that are not themselves motile [21]. EMT is not only crucial for embryogenesis, but this event is a prerequisite for the progression of carcinogenesis as well. Because we found that Cyr61 is essential for the morphological alteration (i.e. EMT) and *in vitro *migration of PDAC cells, we sought to determine if Cyr61 modulates the expression of EMT and stem cell molecular markers. To do so, expression profiles of different epithelial and mesenchymal markers were evaluated in Panc-1^Cyr61+ ^and Panc-1^KOCyr61 ^cells. In the absence of Cyr61, the epithelial markers (i.e., E-cadherin, β-catenin) expressions significantly increased, while the expressions of mesenchymal/stem cell markers (i.e., Vimentin, Notch-1, Oct-4, ABCG2 and CD44) markedly decreased **(Figures **5A**and **5B**)**. We found consistent results when Cyr61 paracrine action was blocked by the addition of Cyr61 antibody (different concentrations) in the media of Panc-1 cell cultures **(Figure **5B**)**. We found consistent results in AsPC-1 cell line (data not included). In order to confirm this phenomenon, Cyr61 negative or minimal expressing BxPC3 cells were exposed to Cyr61 recombinant protein (100 ng/ml)[22] for 48 h and morphology as well as molecular markers of EMT and stemness were characterized. We found that addition of Cyr61 in the culture media markedly enhanced the EMT and stemness in these cells **(Figure **6**)**.

### Cyr61/CCN1 over expressed in Side populations/tumor-initiating cells/cancer stem cells of pancreatic cancer cells

**Figure 7:**
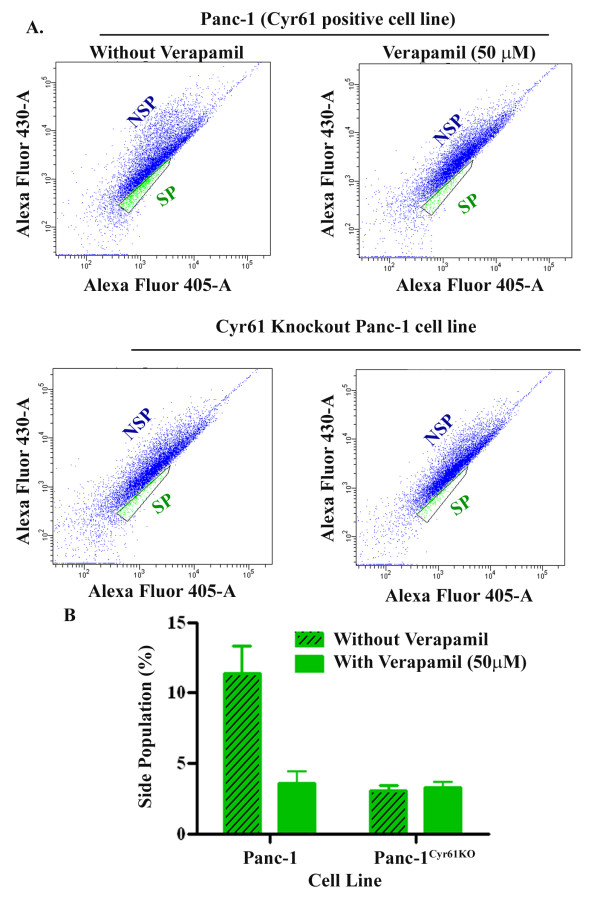
**Flow cytometric analysis of Panc-1^Cry61+ ^and Panc-1^KOCyr61^cells labeled with Vybrant DyeCycle Violet stain**. **(A)**. Side population (SP) cells were detected in Panc-1^Cry61+ ^cells in absence of verapamil (upper left panel). Significant reduction in SP cells was observed in the presence of 50μM verapamil (upper right panel). Lower left and right panels are showing the traces of SP cells in Panc-1^Cry61+ ^and Panc-1^KOCyr61^cells in the absence and presence of 50μM verapamil, respectively. Results are representative of three independent experiments. The SP cells are outlined in the figure. **(B)**. SP cells are shown in a bar diagram as a percentage of the total cell population.

**Figure 8:**
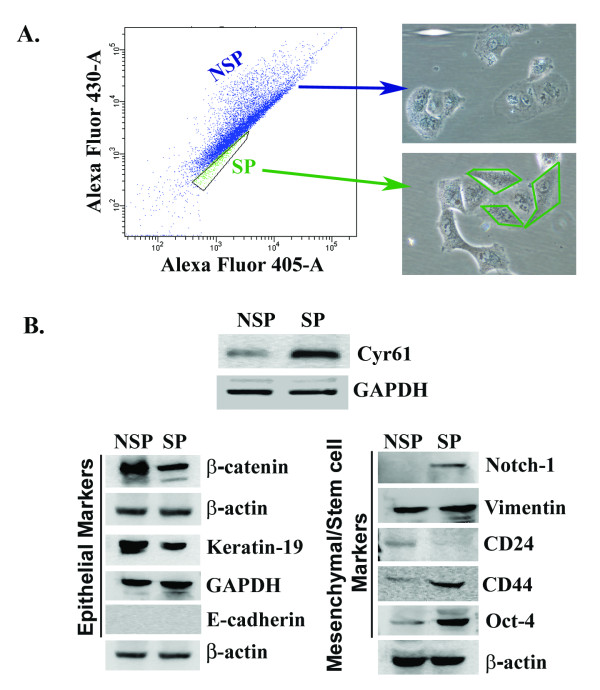
**Morphological and molecular characterization of the SP cells of Panc-1**. **A**. Panc-1 cells were stained with dye-crystal-violet (DCV)-488 Alexa and analyzed by flow cytometry. The representative images were depicted from three independent experiments. The photomicrographs shown in the right panels represent the morphology of SP and NSP cells. **B**. Western blots analysis of Cyr61 and various epithelial, mesenchymal and stem cell markers in SP and NSP cells.

Recently, different laboratories have characterized side population (SP) of pancreatic cancer cells [23,24]. The SP cells are either isolated from cell lines or xenograft tumor cells. The SP is enriched with cancer stem cells, forms tumors in xenograft models and exhibits chemoresistance. Our goal was to examine the status of Cyr61 in the SP of a pancreatic cancer cell line, Panc-1. To do so, first, SP cells were isolated from Panc-1^Cyr61+ ^and Panc-1^KOCyr61 ^cells using a dye-cycle-violet (DCV)-488 Alexa stained Panc-1 cells using a flow cytometry technique [24]. DCV negative cells are considered SP cells and stained cells are NSP cells **(Figure **7A**)**. Consistent with previous works [23,24], our studies demonstrate that there are about 10-15% SP cells in Panc-1 and this population can significantly be reduced by several folds in the presence of Verapamil **(Figure **7A**and **7B**)**, an inhibitor of ABC transporter and known to inhibit side population in various cancer cells [25]. Moreover, negligible percent (less than 3%) of SP cells were detected in Panc-1^KOCyr61 ^cells and these cells are Verapamil insensitive **(Figure **7A**and **7B**)**. Finally, after isolation, both SP and non-SP cells were grown for the determination of Cyr61, epithelial and mesenchymal/stem cell markers using Western blotting. SP cells are morphologically spindle-shaped **(Figure **8A**)**, and over express Cyr61 along with CD44, Notch-1 and Oct-4. CD24, Keratin-19 and β-catenin are down regulated in SP cells **(Figure **8B**)**. Blocking the expression of Cyr61 by shRNA or a Cyr61 neutralizing antibody reduces the expression of stemness markers (i.e., CD44, Notch-1 and Oct-4) (data not shown). Collectively, the data is consistent with previous studies [23,24] indicating that SP cells are enriched with stem cell properties. Cyr61 may play critical role in the formation of SP in Panc-1 cell lines.

### Tumor-initiating capability of Cyr61/CCN1 positive Panc-1-SP cells can be blocked by silencing Cyr61

**Figure 9:**
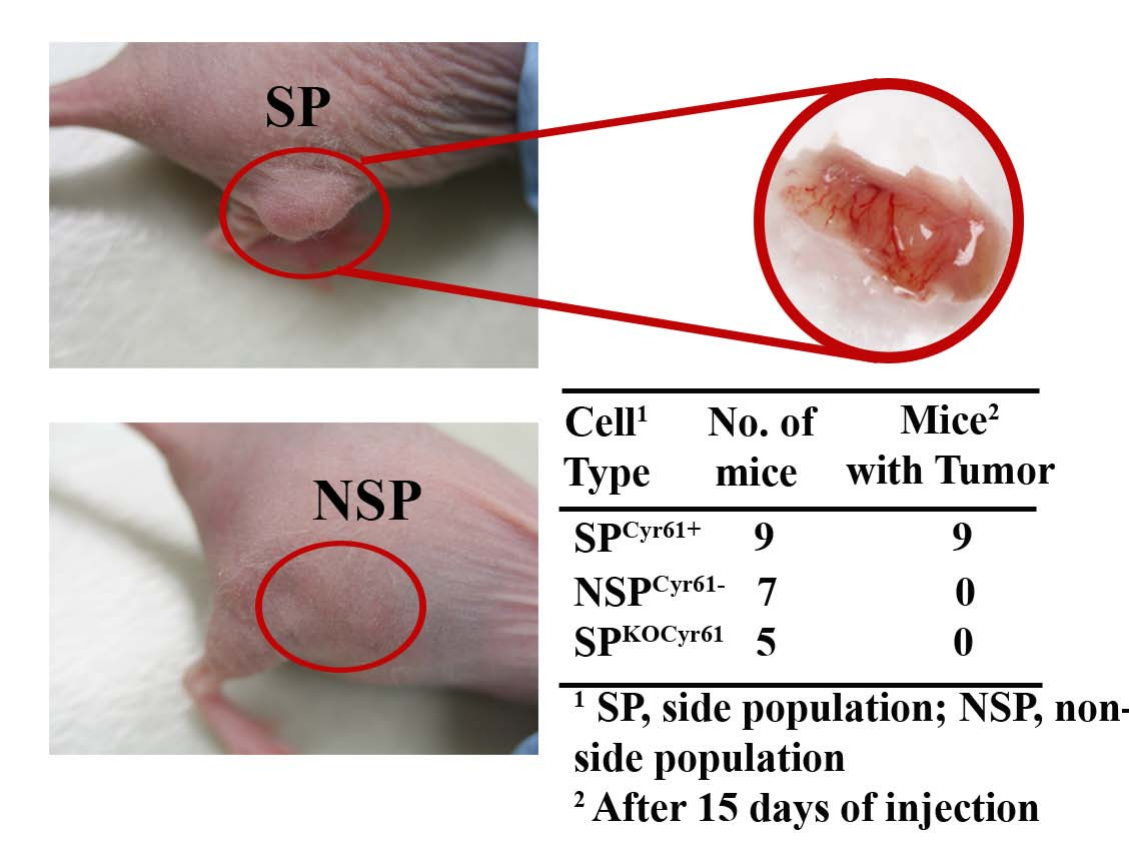
***In vivo *tumorigenicity**. SP, NSP and SP^KOCyr61 ^cells were grown in DMEM with 10% FCS. ~5 × 10^4 ^cells were injected *s.c*. into the right real flank of nude mice and tumor growth was monitored. The number of mice with tumors in each experimental set-up has been presented in the table.

To observe the *in vivo *tumor growth potential of SP cells, both SP and NSP cells (5 × 10^4^) cells with Matrigel were injected into the rear flank of the each mouse (5-9 mice/exp). At the site where the SP cells were injected, nine of 9 mice demonstrated tumor formation 10-15 days post injection with evidence of angiogenesis **(Figure **9**)**. The xenograft tumors overexpressed Cyr61 and other markers. SP-tumors were developed in all nine mice 20 days post injection. However, tumors were not detected in the animals injected with NSP cells **(Figure **9**)**. Tumor formation was detected in two out of 5 mice 30 days post the injection of unsorted Panc-1 cells (data not shown). Our next goal was to find the role of Cyr61 in tumor formation of SP cells in the xenograft model. To do so, Cyr61 was silenced in SP cells by stable transfection of a Cyr61-shRNA containing retroviral. SP cells and Cyr61 silenced SP cells (SP^KOCyr61^) were injected into nude mice and tumor growth was evaluated 15-20 days post injection. Like NSP, SP^KOCyr61 ^cells were unable to form tumor in the xenograft even after 20 days post injection **(Figure **9**)**. Collectively, these studies suggest that Cyr61 may play a critical role in stemness and tumor initiating capability in a population of pancreatic cells.

### Regulation of microRNA (miRNA) by Cyr61 in Panc-1 cells

**Figure 10:**
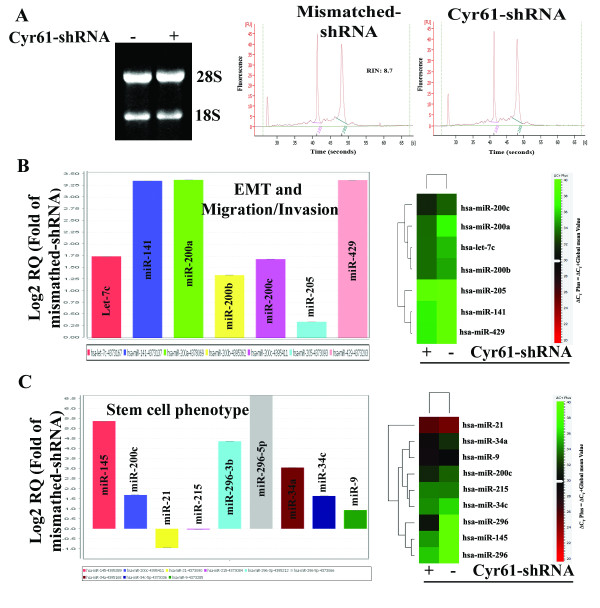
**Expression profiling of microRNA in mismatched and Cyr61-shRNA transfected Panc-1 cells**. **C**. The left panel shows the 28S and 18S bands of total RNA extracted from mismatched-shRNA and Cyr61-shRNA transfected Panc-1 cells. The middle and right panels depict the electropherograms of total RNA validating its integrity. **D**. After data set normalization, the Log2 of the relative quantification for specific miRNAs associated with inhibition of EMT and migration/invasion was plotted as a bar graph. The right panel shows a heat map comparing the average fold-changes in microRNAs with significantly higher (red) or lower (green) expression in Cyr61-shRNA transfected Panc-1 cells in comparison to mismatched-shRNA transfected Panc-1 cells as determined by miRNA Array analysis. Then the signal was globally normalized and Average Linkage Clustering was performed using a Euclidean Correlation. Clustering was visualized using the program provided by Applied Biosystems, Foster City, CA. **E**. Profiling of microRNAs associated with stem cell traits.

To gain further insight into the factors critically regulated by Cyr61 in pancreatic cancer cells, we established quantitative miRNA expression profiles of candidate markers in Cyr61 knockout Panc-1 cells. Prior to microarray analysis, the quality of each RNA sample was verified by determining the quality of RNA **(Figure **10**)**. The miRNA expression analysis was carried out in three independent culture samples and we performed pair wise comparisons of each culture. Data was converted into log2 ratios comparing levels of miRNA expression in Cyr61^+ ^and Cyr61 knockout Panc-1 cells. We observed a dramatic alteration in the miRNA expression profiles in Cyr61 knockout Panc-1 cells and we identified miRNAs that are critically involved in EMT, migration and invasion, and stemness **(Figures **10B**and **10C**)**. In particular, we noticed an increased in the miR-200 family (miR-200a, miR-200b, miR-200c, miR-141 and miR-429) in Panc-1^KOCyr61 ^cells **(Figure **10B**)**. This family of miRNAs is known to regulate EMT and tumor aggressiveness [26]. Furthermore, the microRNAs, which are associated with the inhibition of stemness [26-28] are upregulated, while those responsible for stem cell generation [29] are down regulated in Cyr61-shRNA transfected Panc-1 cells **(Figure **10C**)**. While future systematic Northern blots and *in situ *hybridization screens in these cells and human tissue samples are required to validate all miRNAs, we corroborated the differential expression by qPCR in these cells and human pancreatic cancer cells (data not shown).

## Discussion

The present studies demonstrate that Cyr61 is an important pancreatic cancer marker and that it plays a novel pathobiological role in the development of PDAC. First, we found that Cyr61 is consistently overexpressed in early precursor lesions and its expression increased with advancing disease. 81-85 percent of PDAC patients' samples show Cyr61 positivity **(Figure **1**)**. Cyr61 expression was also detected in different PDAC cell lines. However, the expression profiles were different among the different cell lines. The aggressive cell lines, in which expression profiles of mesenchymal/stem cell molecular markers are predominant, exhibit more Cyr61 expression compared to less aggressive types **(Figures **2**and **3**)**. Second, we observed that Cyr61 plays a critical regulatory role in EMT, stemness and migration of pancreatic cancer cells **(Figures **4, 5, 6, 7, 8**)**. Third, we found that a Cyr61-positive side population (SP) of Panc-1 cells is tumorigenic in a xenograft model and prevention of Cyr61 expression by RNAi (shRNA) in SP cells suppresses the tumor growth ability of these cells drastically **(Figure **9**)**. Finally, depletion of Cyr61 expression by RNA*i *in Panc-1 cells prevented multiple miRNA expressions that are known to regulate EMT, stemness and migration (**Figure **10**)**. These results, collectively, indicate that the activation of Cyr61 signaling in pancreatic cancer cells is one of the early events and is critically linked to the aggressive behavior of these cells including EMT induction and reprogramming of stemness in these cells.

Multiple studies from various laboratories have suggested that PDAC mainly arises from pancreatic ducts through sequential, atypical histological preneoplastic changes (also known as pancreatic intraepithelial neoplasms; PanINs) leading to the development of well to poorly differentiated cancers [30-32]. These sequential transformation events require some oncogenic mutations (i.e. *K-ras *and p53) [33] and/or aberrant expressions of certain genes, reorganizing many cellular features linked with cellular growth and survival. These include EGFR (epidermal growth factor receptor) [34,35], Notch-1[36] and most importantly, the Hedgehog (Hh) signaling pathway [37,38]. We show that Cyr61 is aberrantly overexpressed in histologically defined precursor lesions (PanINs; PanIN-1A-PanIN-3) and its mRNA and protein levels are markedly elevated in varying grades of PDAC specimens compared to adjacent normal tissues where its expression was almost undetected. Cyr61 expression was also differentially expressed in different pancreatic cancer cell lines depending on their morphological and pathobiological behavior. Since several lines of evidence support the role of Cyr61 in promotion as well as progression of various cancers, the present studies highlight the importance of aberrant expression of Cyr61 in pancreatic carcinogenesis.

Cyr61 showed increased expression in metastatic lesions in a clinically relevant model of pancreatic adenocarcinoma. This increase suggested that the interaction between Cyr61 and α_v_β_3 _may promote formation of peritoneal metastases [17], yet its role in PDAC still remains poorly understood. The acquisition of a metastatic phenotype by cancer cells is a complex, multistep process. This process includes EMT followed by stemness, migration and invasion [39,40]. Loss of Cadherin expression or function [41], aberrant regulation of β-catenin [42], Notch-1 [43] and stemness [40] are hallmarks of EMT. Our studies show that Cyr61, which, when overexpressed in PDAC and its precursor lesions **(Figure **1**)**, promotes EMT possibly through down-regulating E-cadherin and its interacting partners such as β-catenin **(Figures **3, 4, 5, 6**)**. In an *in vitro *setup, we have also demonstrated that stemness-like state can be achieved in the presence of Cyr61 through the regulation of multiple stemness traits including ABCG2, Notch-1, Oct-4 and CD-44 in pancreatic cancer cells. Moreover, we also found that Cyr61 is a positive regulator of the pancreatic cancer cell migration, one of the hallmarks of cancer that leads cancer cells to invade for metastatic growth to the distant organs. Collectively, from these experiments, we assumed that the activation of Cyr61/CCN1 may play a critical role in the reprogramming and maintenance of cancer stemness/tumor initiating cells through EMT process in parental counterparts, and subsequently enhance the migration of these cells. We are not aware though of any data that support this idea (i.e., EMT instigates stemness) other than the reports of Mani *et.al*. [40].

The above perception of Cyr61/CCN1 is further strengthened with our side-population (SP) studies **(Figures **7**and **8**)**. These studies showed that a side population (SP) of pancreatic cancer cells, which has mesenchymal/stemness features, produced a *sc *tumor with overexpressed Cyr61in nude mice within a brief period **(Figure **9**)**. RNA*i*-based nullification of Cyr61 in SP cells reverses the cellular and molecular features of SP cells, and they behave like the non-side population (NSP). In addition, Cyr61 knockout cells are unable to develop tumor xenograft tumors in nude mice. Taken together, these studies suggest that Cyr61 seems to participate in SP generation and SP-tumorigenicity as well. However, the molecular events that are associated with this distinctive process are uncertain. It will be, therefore, interesting to define how Cyr61 deficiency regulates reprogramming by promoting mesenchymal to epithelial transition in pancreatic cancer cells and what specific factor(s) are crucial for leading to a side population state under the influence of Cyr61.

Multiple studies have shown an analogous connection between cancer progressions related events and the expression profiles of miRNAs, an abundant class of non-protein-coding RNAs that function as negative regulators of diverse functional genes [44]. Recent studies have shown that miRNA mutations, mis-expression and malfunction of miRNA machinery correlate with various human cancers' development and progression [44]. In particular, miRNAs of miR-200 family appear to play a critical role in the regulation of EMT and tumor aggressiveness [26] and eventually may play a role in generating cells with the properties of stem cells [40]. Furthermore, from different miRNA profiling experiments it is evident that miRNAs can act as stemness regulators [45]. We have shown here that Cyr61 regulates some of the above mentioned miRNAs that are associated with EMT, stemness and migration/invasion activities **(Figure **10**)**. Therefore, these studies raise the possibility that Cyr61-induced EMT, stemness and migration activity may be driven by the regulation of miRNAs. However, further studies will certainly be required to establish the hypothesis and *in vivo *significance of the cell culture findings, which are in progress.

## Conclusions

**Figure 11:**
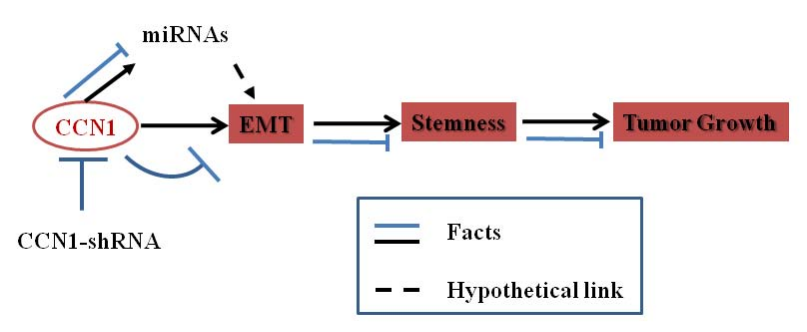
**Diagrammatic illustrations of Cyr61/CCN1 signaling involved in EMT, stemness and development and progression of pancreatic cancer**. The studies speculate that multiple miRNAs, which are actively participate in different phases of carcinogenesis, are participated in Cyr61/CCN1-based carcinogenesis.

In conclusion, these studies, as depicted in **Figure **11, identify Cyr61/CCN1 as a critical regulator of pancreatic carcinogenesis by making an essential contribution in the development of aggressive phenotypes (i.e., EMT followed by stemness) of this disease. These contributions may potentiate through the regulation of miRNAs. Given that Cyr61 activity is crucial for pancreatic cancer cell growth and progression, targeting the Cyr61 pathway may be an attractive therapeutic avenue in PDAC.

## Materials and methods

### Reagents and antibodies

Human polyclonal anti-rabbit Cyr61 antibody was purchased from Santa Cruz Biotechnology (Santa Cruz, CA). Monoclonal anti-mouse GAPDH antibody was purchased from Applied Biosystems (Foster City, CA). Mouse monoclonal Vimentin antibody was obtained from Lab Vision (Fremont, CA), mouse monoclonal anti-β-catenin and rabbit polyclonal Keratin-19 were purchased from BD Transduction Laboratory (San Jose, CA), rabbit polyclonal anti-human Oct-4 and CD44 were purchased from Cell Signaling (Boston, MA). Rabbit polyclonal anti-human Notch-1, mouse monoclonal anti-human CD24, polyclonal goat anti-rabbit IgG-HRP and monoclonal goat anti-mouse IgG-HRP were purchased from Santa Cruz Biotechnology (Santa Cruz, CA). Cyr61 recombinant protein was purchased from Thermo Fisher Scientific (Waltham, MA). pSilencer™ 5.1-U6 retroviral vector and siPORT™ XP-1 transfection agent were obtained from Applied Biosystems (Foster City, CA). Matrigel was purchased from BD Biosciences (Bedford, MA). All other chemicals were obtained either from Sigma (St. Louis, MO) or Fisher Scientific (Houston, TX).

### Tissue samples

Archived, 4% formalin-fixed, paraffin-embedded primary pancreatic adenocarcinoma samples and chronic pancreatitis tissue samples were obtained from the Department of Surgical Pathology of VA Medical Center. The pancreatic tissue array containing adjacent normal (NP), chronic pancreatitis (CP) and different grades of ductal adenocarcinoma (Gr-I, Gr-II and Gr-III) were evaluated in this study. The tissue arrays were purchased from Cybrdi Inc (Frederick, Maryland).

### Cell Culture

All Pancreatic cancer cell lines (i.e., BxPC-3, AsPC-1, Capan-1, and Panc-1) were purchased from American Type Culture Collection (ATCC, Manassas, VA). The cell lines were cultured in Dulbecco's modified Eagle's medium (Sigma, St Louis, MO) supplemented with 10% fetal bovine serum (Hyclone, Logan, UT), 2 mM glutamine, 100 units/ml penicillin and 100units/ml streptomycin (Sigma) at 37°C incubator in the presence of 5% CO_2_. Ampho-pak 293 packaging cell line was purchased from Clontech (California, USA), and was maintained in high glucose DMEM containing 10% FBS. Cells were used for the experiment between four and six passages.

### Immunohistochemistry

The Immunohistochemistry was performed on 4% formalin fixed-paraffin-embedded tissue sections according to our previous method [46,47]. Briefly, tissue sections were deparaffinized in Xylene, rehydrated in different grades of alcohol, washed with PBS and blocked with tissue blocker (Zymed Laboratories, CA) for 10 minutes and immunostained by polyclonal human anti-rabbit Cyr61 antibody (1:300) overnight. The clinical stages obtained from database were reviewed and reconfirmed by a pathologist using adjacent hematoxylin and eosin stained slides. The sections were imaged with a Leica photomicroscope. All samples were used according to VA Medical Center and University guidelines after receiving Institutional Review Board approval.

### RNA Extraction, cDNA Synthesis, and Probe Preparation

Total RNA extraction was essentially the same as that previously described [46,47]. cDNA synthesis and probe preparation were done according to the method described by Banerjee et al [46].

### *In Situ *Hybridization

*In situ *hybridization for Cyr61 mRNA expression was performed on formalin-fixed, paraffin embedded tissue sections according to the method that is described earlier by us [48]. Briefly, the paraffin sections were dewaxed in xylene, rehydrated through different grades of alcohol and digested with proteinase K followed by post-fixation in 10% formaldehyde solution at room temperature. The sections were thoroughly washed with RNAse-free water and incubated with Digoxigenin (DIG)-labeled PCR generated Cyr61 specific non-radioactive probe (250 ng/ml) overnight at 37°C in a humidified hybridization chamber. The hybridized probe was detected using alkaline phosphatase-conjugated anti-DIG antibody (InnoGenex) and visualized with chromogen combination 5-bromo-4chloro-3-indolyl phosphate NBT.

### Northern blot Analysis

For Northern blotting, an established method previously reported by us was used [49]. Briefly, Total RNA (10 μg) of each sample was separated by formaldehyde/agarose gel electrophoresis and transferred to a nylon membrane. The membranes were hybridized with nonradioactive digoxigeninlabeled, PCR-generated probes. Glyceraldehyde- 3-phosphate dehydrogenase (GAPDH) was used as a loading control. Relative expressions of mRNA were calculated by densitometric analyses using One-dimensional Image Analysis Software version 3.6 (Eastman Kodak Co., Rochester, NY).

### Quantitative real-time PCR

Briefly, total RNA was extracted from different pancreatic cancer cell lines using TRIZOL (Invitrogen, Carlsbad, CA). cDNA was prepared from total RNA by using Taqman Reverse Transcription kit. Real-time PCR was performed from cDNA products using Taqman universal PCR and Taqman assay kit by Applied Biosystem Step One real-time PCR system. C_T _values for Cyr61 were normalized to human GAPDH by subtracting the average C_T _value for each sample. Relative quantification (RQ) values for Cyr61 in each sample were determined using the 2^-ΔΔCT ^method [47]. Each PCR reaction was performed in triplicate.

### Western Blot Analysis

Cell lysates from different pancreatic cell lines were prepared for Immuno-Western blotting according to our previous method [46]. Briefly, cells were washed with phosphate-buffer saline (PBS) and lysed in RIPA buffer (50 mM tris-Cl, pH 8.0, 150 mM NaCl, 1% Nonidet P-40, 0.1% sodium dodecyl sulfate) containing the protease inhibitors, 0.5 mM phenylmethylsulfonyl fluoride, 1μM leupeptin, 1μM aprotinin. The lysates were centrifuged at 18000 rpm for 60 min at 4°C. Equal amounts of protein (50 μg), as determined by Coomassie blue reagent assay (Bio-Rad, Richmond, CA), were subjected to 10% SDS-PAGE, and the gel-fractionated proteins were transferred to nitrocellulose membranes (Bio-Rad) and reacted with appropriate antibodies. Signals were detected with Super Signal ULTRA chemiluminescent substrate (Pierce, Rockford, IL) by using one dimensional Image analysis, version 3.6 (Eastman Kodak Co., Rochester, NY).

### Retroviral production and transduction of cells

Human Cyr61-shRNA primers were designed using vector NTI software from Invitrogen. The shRNAs sequences of human Cyr61 are: shRNA-1, forward: 5'- GAT CCG GGA AAG TTT CCA GCC CAA TTC AAG AGA AGT TGG GCT GGA AAC TTT CCC TTT TTT GGA AA -3'; reverse: 5'- AGC TTT TCC AAA AAA GGG AAA GTT TCC AGC CCA ACT TCT CTT GAA TTG GGC TGG AAA CTT TCC C -3'; shRNA-2, forward: 5'- GAT CCG CCC AAC TGT AAA CAT CAG TTC AAG AGA CAC TGA TGT TTA CAG TTG GGC TTT TTT GGA AA -3'; reverse: 5'- AGC TTT TCC AAA AAA GCC CAA CTG TAA ACA TCA GTG TCT CTT GAA CTG ATG TTT ACA GTT GGG C -3'. Recombinant clones of Cyr61shRNA are produced using pSilencer™5.1-U6 Retro-viral Vector from Ambion (Austin, TX) as per the protocol described in the instruction manual. Recombinant clones were confirmed by sequencing using sequencing primers. Scrambled control provided in the kit was used as a control. Briefly, Cyr61shRNA is transfected in Amphopak ™293 packaging cell line using siPORT™ XP-1 transfection agent. Supernatant containing viral particles was collected after 72 h. ~60% Panc-1 cells were infected with Cyr61-shRNA containing viral supernatant or scrambled control with different dilutions (from 1:10^1 ^to 1:10^5^) and incubated for 72 hrs. Stable cell lines were prepared after prolonged puromycin treatment. Stable cells were then cultured in regular DMEM media with 10% FBS and harvested for western or northern blot analysis to check the transfection efficiency.

### MicroRNA (miR) Array Analyses

For miRNA array experiments, total RNA was isolated from mismatched-shRNA and Cyr61-shRNA transfected Panc-1 cell lines by Trizol method as described in the prior section. The integrity of total RNA was assessed by running RNA sample on a denaturing agarose gel stained with ethidium bromide. The 2:1 ratio of 28S and 18S are considered as a good indication of intact RNA. cDNA was synthesized using Megaplex RT PCR kit (Applied Biosystems, Foster City, CA) for Array A, which contains 384 stem-looped reverse transcription primers specifically binds to miRNAs. Briefly, 500 ng of total RNA and 4.5μl of RT reaction mix in a total volume of 7.5μl were processed for cDNA preparation at the following cycle conditions: 16°C for 2 min, 42°C for 1 min and 50°C for 1 min for total of 40 cycles followed by 85°C for 5 min and bringing the contents to 4°C. The human miRNA array system (Array A) (Applied Biosystems, Foster City, CA) was used for the detection and quantification of miRNA in mismatched-shRNA and Cyr61-shRNA transfected Panc1 cells. This miRNA-array kit consists of four plates of plate A and four plates of plate B which contain around 384 miRNAs including four internal controls. For this we used 6μl of cDNA synthesized by using Megaplex RT and 450 μl of TaqMan universal PCR master mix in a total of 900 μl of reaction volume and 100 μl of the reaction mixture was loaded into each port provided in the card (which has 8 ports for each card). The cards were run in Applied Biosystems Real-time PCR system (7900 HT) by selecting relative quantification (ΔΔCt) at following conditions: 95°C for 10 min, 95°C for 1 min and 60°C for 1 min for total of 40 cycles. All the samples were run in duplicates. Finally, all the raw data from each card was retrieved from the 7900HT machine and was run on Data Assist Software ver.1.2 (Applied Biosystems, Foster City, CA). The mean values for RQ (which is fold values of Cy61-shRNA transfected compared to mismatched-shRNA transfected panc1 cell lines) were used to plot the bar diagrams and heat map clusters.

### In vitro Boyden chamber migration assay

The chemotaxis assay was conducted using a modified Boyden chamber technique as described previously [46]. Briefly, Panc-1 cells (20,000 cells/well), which were either infected with viral particles containing shRNA (Cyr61/scrambled) or treated with Cyr61 neutralizing antibody for 48 h, were added to the upper chambers of the Boyden chamber (8 micron) containing DMEM with 1% FBS. Lower chamber was loaded with DMEM with 10% FBS. Cells were allowed to migrate for 24 hrs. The migratory cells that were attached on the undersurface of Boyden chamber were stained with crystal violet solution for 10 min. Inserts were washed with tap water and then air dried for 30 minutes. Crystal violet stained cells were solubilized with 10% acetic acid and optical density is quantitated in Microplate reader at 600 nm. Three wells were examined for each condition and the experiments were repeated three times.

### Isolation of side population (SP) by Flow cytometry

The side population/stem cells from Panc-1 cell line were isolated according to the previous methods with brief modifications [24]. Briefly, ~80 percent confluent cells were incubated with dissociation solution (Sigma Chemical Co. St. Louis) for 15 min at 37°C, and dissociated cells were counted and transferred to a 5 ml tube. Washed twice with pre-warmed DMEM containing 10% FBS. Finally, cells were resuspended in same media at concentration of 1 × 10^6^cells/100μl. Vybrant-Violet solution (10μM) and Verapamil (50 μM) solution were added into the sample and incubated at 37°C for 90 min. After incubation, cells were centrifuged, and resuspended in ice-cold 1 × PBS, pH 7.4. 2μg/ml propidium iodide was added immediately before flow cytometry analysis to exclude dead cells. SP cells were identified, sorted, and analyzed on a BD FACS Aria SORP flow cytometer (BD Biosciences) using ~405 nm excitation and 440 nm emission. Sorted cells (i.e., SP and Non-SP) were washed in serum-free medium and then cultured in DMEM with 10% FCS for several days in 5% CO_2 _at 37°C. The cell lysate was extracted from semi-confluent cells for the analysis of different epithelial, mesenchymal and stem cell markers by Western blot analysis using specific antibodies.

### Sorted cell implantation in athymic nude mice

Male athymic nude mice (*nu/nu *genotype), 6 to 8 weeks old, were obtained from Charles Rivers (Wilmington, MA) and acclimated in our facility for 1 week. The animal studies were conducted according to the approved Guidelines of the Animals Care and Use Committee of Kansas City VA Medical Center. Sorted cells (SP and NSP) were grown in DMEM with 10% FCS using the same procedure as described herein. Cells (5 × 10^4^) were injected *s.c*. into the right rear flank of each mouse (5-9 mice per group) and tumor growth was monitored after 2^nd ^days of injection and continued up to 21 days.

### Statistical Analysis

The results of each experiment were the representative of at least three sets of experiments performed in triplicate. All data were expressed as the mean ± SEM. Statistically significant differences between groups were determined by using the non paired Student's two-tailed *t*-test. A value of P < 0.05 was considered statistically significant.

### Author details

^1^Cancer Research Unit, VA Medical Center, 4801 Linwood Blvd, Kansas City, Missouri 6 4128, USA. ^2^Division of Hematology and Oncology, 2330 Shawnee Mission Pkwy, Westwood, Kansas 66205, USA, ^3^Department of Anatomy and Cell Biology, 3901 Rainbow Blvd, Kansas City, KS 66205, University of Kansas Medical Center, 3901 Rainbow Blvd, Kansas City, Kansas 66160, USA.

## Competing interests

The authors declare that they have no competing interests.

## Authors' contributions

SKB and SB initiated the project and designed the studies. IH and MM performed all the transfection studies and related experiments including Western blots, immunohistochemistry and *in situ *hybridization. KD, MM, IH and SM were involved in *in vitro *and *in vivo *side population studies. AD has assisted with the in vitro studies and EMT analysis using Western blotting. SM and IH with the help of PJV wrote the initial draft of the manuscript. DM, a pathologist, analyzed all stained slides. All authors helped in discussing, reading and proofreading the final manuscript.
